# EXPLORING HOW CARE PROVIDERS TALK ABOUT COMMUNICATION DIFFICULTIES WITH ETHNIC MINORITY PATIENTS

**DOI:** 10.1093/geroni/igz038.841

**Published:** 2019-11-08

**Authors:** Pernilla Ågård

**Affiliations:** Deparment of Sociology, Uppsala University, Uppsala, Sweden

## Abstract

Previous research into cross-cultural interactions in health care settings shows that care providers experience communicating with elderly ethnic minority patients as problematic. According to the social constructionist framework upon which this presentation draws, people negotiate the characteristics they ascribe to the world around them through talk. It is against this backdrop that the presentation – which is based on a focus group study with end-of-life care providers (n=60) – sets out to explore how care providers talk about communication difficulties with elderly ethnic minority patients. The presentation demonstrates how the study of communication difficulties can illustrative the challenges of cross-cultural interaction in end-of-life care settings. Through the attention on how communication difficulties are discussed, this presentation shifts the focus from the elderly ethnic minority patients and the reasons for why they are experienced as problematic when it comes to communication, to the actual process where these problems are negotiated.

